# Germline and somatic genetic alterations in two first‐degree relatives with appendiceal low‐grade mucinous carcinoma peritonei

**DOI:** 10.1002/ccr3.3338

**Published:** 2020-09-29

**Authors:** Mary Caitlin King, Carlos Munoz‐Zuluaga, Panayotis Ledakis, Kimberley Studeman, Michelle Sittig, Vadim Gushchin, Armando Sardi

**Affiliations:** ^1^ The Institute for Cancer Care Mercy Medical Center Baltimore MD USA; ^2^ Department of Pathology Mercy Medical Center Baltimore MD USA

**Keywords:** appendix cancer, genetic analysis, hyperthermic intraperitoneal chemotherapy, next‐generation sequencing, pseudomyxoma peritonei

## Abstract

Comparing genetic mutations of first‐degree relatives with appendiceal pseudomyxoma peritonei may explain clinical outcomes and disease pathogenesis. Molecular profiling of mucinous tumors may identify improved treatments to traditional chemotherapy.

## Graphical abstract

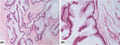

## 1 INTRODUCTION

In this rare case of appendiceal pseudomyxoma peritonei in first‐degree relatives, shared novel germline mutations (RAD51C, FH), and somatic mutations (KRAS, GNAS, TSC1) may account for similar disease presentation and elucidate disease pathogenesis. Differences in somatic mutations may explain their variant clinical outcomes and elucidate higher efficacy targeted therapies.

Appendiceal cancer is a rare, lethal disease, occurring in <1% of appendectomies.^1^ Most are mucinous and frequently (20%) present with pseudomyxoma peritonei (PMP), a syndrome characterized by mucinous ascites and diffuse peritoneal disease.^2^, ^3^, ^4^ PMP is classified as either low‐grade mucinous carcinoma peritonei (LGMCP) or high‐grade mucinous carcinoma peritonei (HGMCP).^5^ Currently, the standard treatment is cytoreductive surgery (CRS) and intraoperative hyperthermic intraperitoneal chemotherapy (HIPEC) with the best outcomes associated with low disease burden and complete cytoreduction.^3^, ^6^, ^7^, ^8^ Despite improvement in modern management, disease recurrence is frequent and associated with significant mortality with 10‐year overall survival ranging from 0% to 46% depending on histopathologic subtype.^9^, ^10^, ^11^, ^12^

Appendiceal mucinous neoplasm (AMN) and PMP pathogenesis are largely unknown. The role of bacteria and various mutations are being investigated, but any association with clinical outcomes is unclear.^13^, ^14^, ^15^, ^16^, ^17^, ^18^, ^19^ The possibility of a germline susceptibility to appendiceal cancer has yet to be fully investigated. To our knowledge, there are four reported familial instances of appendiceal neoplasms, none of which had complete genomic testing (germline and somatic) on both patients.^16^, ^20^, ^21^, ^22^ Understanding appendiceal LGMCP pathogenesis is essential in early diagnosis and development of effective treatments.

We report the first case of two first‐degree relatives (mother/daughter) with LGMCP originating from a low‐grade appendiceal mucinous neoplasm (LAMN), but with different treatment response. Germline and somatic analysis was performed for insight into the molecular drivers of appendiceal LGMCP.

## 2 CASE REPORTS

### 2.1 Case 1: Mother

A 66‐year‐old Caucasian female with history of hypertension, congestive heart failure, and hypothyroidism presented to her gynecologist with abdominal fullness and rectocele and underwent workup for a hysterectomy. Abdominopelvic CT revealed massive complex ascites with enhancing nodular components and omental caking concerning for malignancy. Paracentesis cytology showed abundant mucin and few entrapped mesothelial cells, consistent with PMP. No definitive malignant cells were seen.

**Figure 1:**
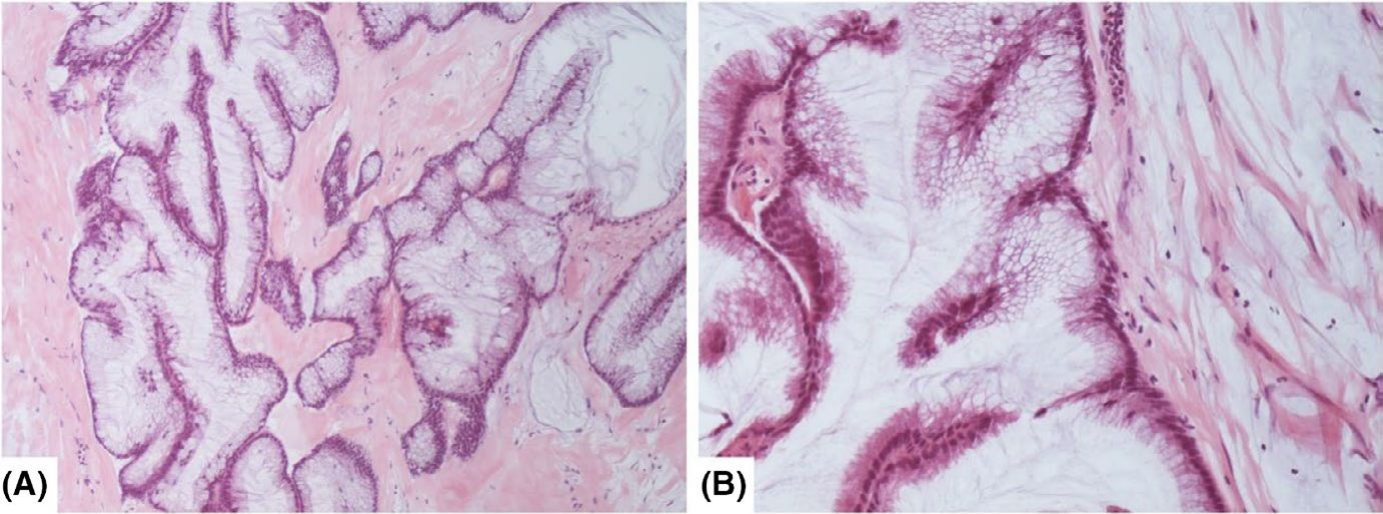
The mother's ruptured appendix after her CRS/HIPEC procedure showed hypermucinous adenomatous epithelium (A. 10X & B. 40X) consistent with a low‐grade appendiceal mucinous neoplasm (LAMN). The daughter had her appendectomy at an outside institution, which was reviewed by our center and showed the same ruptured LAMN

**Figure 2:**
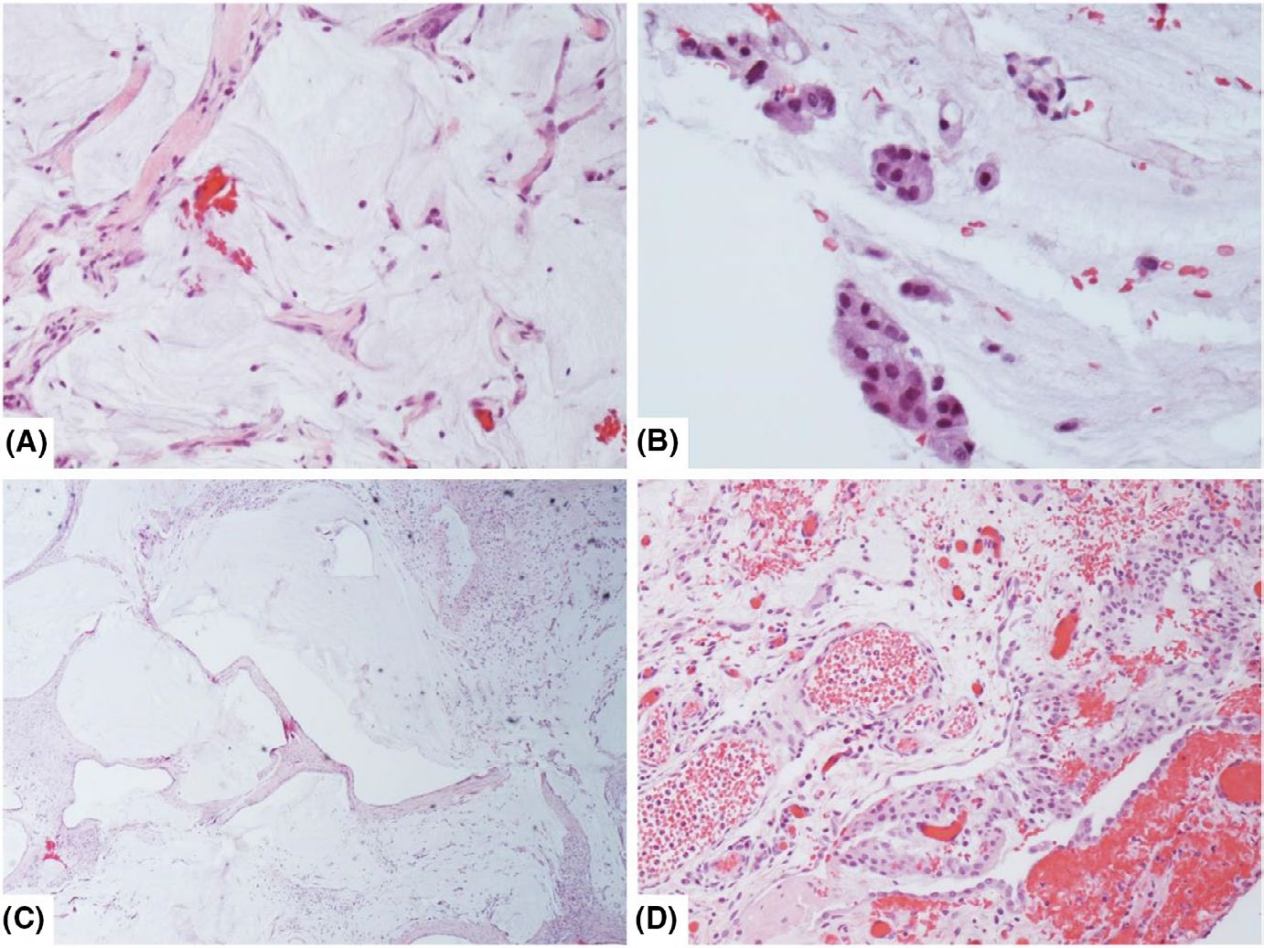
Peritoneal dissemination of low‐grade mucinous carcinoma peritonei (LGMCP, previously classified as disseminated peritoneal adenomucinosis [DPAM]) in peritoneal implants from the mother (A & B) and daughter's (C & D) CRS/HIPEC procedures. Although both cases had organizing mucin and adenomatous epithelium (A. 20X, B. 40X, and C. 4X), the daughter's histopathology had more extensive tissue involvement, more abundant mucin, and a stronger immune response with mesothelial hyperplasia, granulation tissue, and fibrosis (D. 20X)

Given the likelihood of appendiceal malignancy, she was referred to surgical oncology. Preoperative CEA and CRP were elevated, while CA 125 and CA 19‐9 were normal. She underwent CRS/HIPEC with mitomycin‐C 64 days after initial diagnosis. Resections included abdominal wall tumor, liver capsules (segments I, III, V‐VIII), bilateral parietal and diaphragmatic peritonectomies, appendectomy, splenectomy, omentectomy, hysterectomy, bilateral salpingo‐oophorectomy, cholecystectomy, low anterior resection, and tumor debulking. Pre/postoperative peritoneal cancer index (PCI) was 39/8, respectively, with completeness of cytoreduction (CC) score of 2. Small bowel mesenteric nodules and membranes presumed to be residual disease were unable to be removed. Postoperative course was uncomplicated and she was discharged after 10 days. Pathology showed LGMCP originating in a LAMN (Figure 1). Adenomatous epithelium and organizing extracellular mucin were present throughout the peritoneal cavity (Figure 2A,B). All lymph nodes (n = 44) were negative for metastatic disease.

The mother is clinically stable at 58 months. Most recent CT was negative for tumor and tumor markers were within normal limits (CEA: 5.7 ng/mL; CA 125:12.3 U/mL; CA 19‐9:11 U/mL) (Table 1).

**Table 1:** Mother and daughter perioperative characteristics

Characteristic	Mother	Daughter	
		1st Surgery	2nd Surgery
Age at diagnosis, y	66	40	‐
Diagnosis	LGMCP	LGMCP	LGMCP
Appendix pathology	LAMN	LAMN	*‐*
Preoperative			
Previous surgery	Paracentesis	Omental biopsy TAH/BSO/R hemicolectomy/CRS	CRS x2
PSS	0	3	3
Pre‐Op CEA, ng/mL	57.4 (↑)	6.9 (↑)	18.6 (↑)
Pre‐Op CA 125, U/mL	10.0 (‐)	37.4 (↑)	7.5 (↑)
Pre‐Op CA 19‐9, U/mL	21.0 (‐)	26 (‐)	55.3 (↑)
Pre‐Op CRP, MG/DL	2.755 (↑)	0.999 (‐)	9.742 (↑)
CRS/HIPEC			
Time to CRS/HIPEC, d	64	76	*‐*
Age at surgery, y	67	41	44
HIPEC agent	Mitomycin‐C (40 mg, 90 min)	Aborted	Mitomycin‐C (40 mg, 90 min)
PCI score (Pre/Post‐CRS)	39/8	Aborted	39/12
CC score	CC‐2	Aborted	CC‐3
Sites of residual disease	Small bowel mesentery	Stomach, porta hepaticus, duodenum, mesentery	Stomach, porta hepaticus, gallbladder
LN status (+/total)	0/44	0/1	0/8
ICU Stay, d	2	0	1
Hospital stay, d	10	5	8
Complications	None	Aborted	Anemia, abdominal rash
Follow‐up			
Adjuvant therapy (duration/cycles)	None	CAPE (7 mo), FOLFOX (seven cycles), XELOX (8 mo), CAPE (4 mo), CAPE/Irinotecan (one cycle)	None
Overall survival, mo	58	46	12
Status	AWD	AWD	AWD
Symptoms	None	Bloating, fullness	None

Abbreviations: AWD, Alive with disease; BSO, Bilateral salpingo‐oophorectomy; CA 19‐9, Cancer antigen 19‐9; CA‐125, Cancer antigen 125; CAPE, Capecitabine; CC‐Score, Completeness of cytoreduction score; CEA, Carcinoembryonic antigen; CRP, C‐reactive protein; CRS, Cytoreductive surgery; FOLFOX, Oxaliplatin with 5‐fluorouracil and folinic acid; HIPEC, Hyperthermic intraperitoneal chemotherapy; ICU, Intensive care unit; LAMN, Low‐grade appendiceal mucinous neoplasm; LGMCP, Low‐grade mucinous carcinoma peritonei; LN, Lymph node; PCI, Peritoneal cancer index; Pre‐Op, Preoperative; PSS, Prior surgical score; TAH, Total abdominal hysterectomy; XELOX, Oxaliplatin with capecitabine.

### 2.2 Case 2: Daughter

A 40‐year‐old Caucasian female presented to her PCP for rapidly increasing abdominal distention and intermittent right lower quadrant pain, <1 year after her mothers’ appendiceal diagnosis. Abdominopelvic CT revealed a small amount of ascites, bilateral ovarian masses, and omental metastases, likely ovarian origin or PMP. CEA and CA 125 were elevated at 99.1 ng/mL and 75.1 U/mL, respectively. Omental biopsy was performed. The patient was counseled for ovarian cancer treatment and underwent a right hemicolectomy, hysterectomy, bilateral salpingo‐oophorectomy, and incomplete debulking by a gynecologist prior to final biopsy results. Pathology was consistent with LGMCP originating in a LAMN. Adenomatous epithelium and mucin were noted throughout the peritoneal cavity. All lymph nodes (n = 23) were negative for metastatic disease.

With an identical diagnosis as her mother, she elected to proceed with CRS/HIPEC by the same surgical team. Preprocedure CEA and CA 125 remained elevated, while CA 19‐9 and CRP were normal. Ninety‐three‐day post initial consult and 76‐day post debulking surgery, CRS/HIPEC was attempted; however, extensive tumor involvement in the lesser curvature of the stomach, porta hepaticus, duodenum, and small bowel mesentery prevented a compete cytoreduction and HIPEC was aborted. A partial palliative cytoreduction was performed, including resection of abdominal wall, omentum, bilateral parietal peritoneum, liver capsules (segments VII‐VIII), and tumor debulking. Postoperative course was uncomplicated and she was discharged after 5 days. Pathology showed LGMCP at all resected sites with one negative lymph node.

She received multiple regimens of palliative 5‐FU‐based chemotherapies with capecitabine, folinic acid with fluorouracil and oxaliplatin (FOLFOX), capecitabine plus oxaliplatin, and capecitabine plus irinotecan with several breaks over a 3 year period. She was reevaluated for CRS/HIPEC upon clinical symptomology and radiographic evidence of disease progression.

Three‐year post aborted CRS/HIPEC, she underwent a second CRS/HIPEC with mitomycin‐C for palliation. Preoperative CEA, CRP, and CA19‐9 were elevated. Extensive tumor and ascites were noted, with pre/postoperative PCI of 39/12 and CC‐3. The stomach, porta hepaticus, and gallbladder had unresectable disease. Postoperative course was uncomplicated and she was discharged after 8 days. Pathology was consistent with LGMCP. All lymph nodes (n = 8) were negative (Figure 2C,D).

The patient has stable, asymptomatic disease at 46‐month post aborted CRS/HIPEC and 12‐month post palliative CRS/HIPEC. Most recent tumor markers were normal (CEA: 4.3 ng/mL; CA‐125: 8.0 U/mL; CA 19‐9: 26 U/mL) (Table 1).

## 3 METHODS

### 3.1 CRS/HIPEC

Preoperative evaluation at our high‐volume CRS/HIPEC center includes physical exam, abdominopelvic imaging, and tumor markers, including CEA (N < 5 ng/mL), CA 125 (N < 35 U/mL), CA 19‐9 (N < 37 U/mL), and CRP (N < 1.00 MG/DL). Intraoperatively, disease burden is assessed using the peritoneal cancer index (PCI) (0‐39).^23^ Resections are performed to reduce tumor to microscopic levels. After CRS, the completeness of cytoreduction score is recorded (CC‐0: no residual tumor, CC‐1: tumor nodules <2.5 mm, CC‐2: tumor nodules 2.5‐25 mm, and CC‐3: tumor nodules >25 mm).^23^ For appendiceal malignancies, HIPEC is performed using the closed technique with 40 mg of mitomycin‐C heated to 41‐42°C for 90 minutes. After perfusion, anastomoses are completed. Postoperatively, patients are transferred to the ICU for the first 24 hours and then to the inpatient unit when stable. Complete details of our management were previously published.^6^

### 3.2 Germline genetic testing

A 3‐generation pedigree and risk assessment was performed for both patients. Invitae Multi‐Cancer Panel^®^ was recommended which assesses 79 genes associated with hereditary cancer. Genomic DNA obtained from the submitted blood sample was analyzed with full‐gene sequencing and deletion/duplication analysis using next‐generation sequencing (NGS) Illumina technology with >99% specificity for base substitutions, insertions, and deletions. Targeted regions are covered with ≥50x depth.

### 3.3 Tumor genetic testing

Appendiceal primary tumors from archived formalin‐fixed, paraffin‐embedded tissue were reviewed by a specialized pathologist and prepared in sections. Comprehensive genomic tumor profiling was performed using FoundationOne^®^ CDx which applies NGS using Illumina HiSeq 4000 platform to identify genomic alterations across 315 cancer‐related genes plus introns from 28 genes often rearranged in cancer. FoundationOne^®^ achieves >99% specificity for base substitutions, indels, copy alterations, and rearrangements. Median exon coverage was 332x and 757x for the mother and daughter, respectively.

## 4 RESULTS

### 4.1 Pedigree & family history

**Figure 3:**
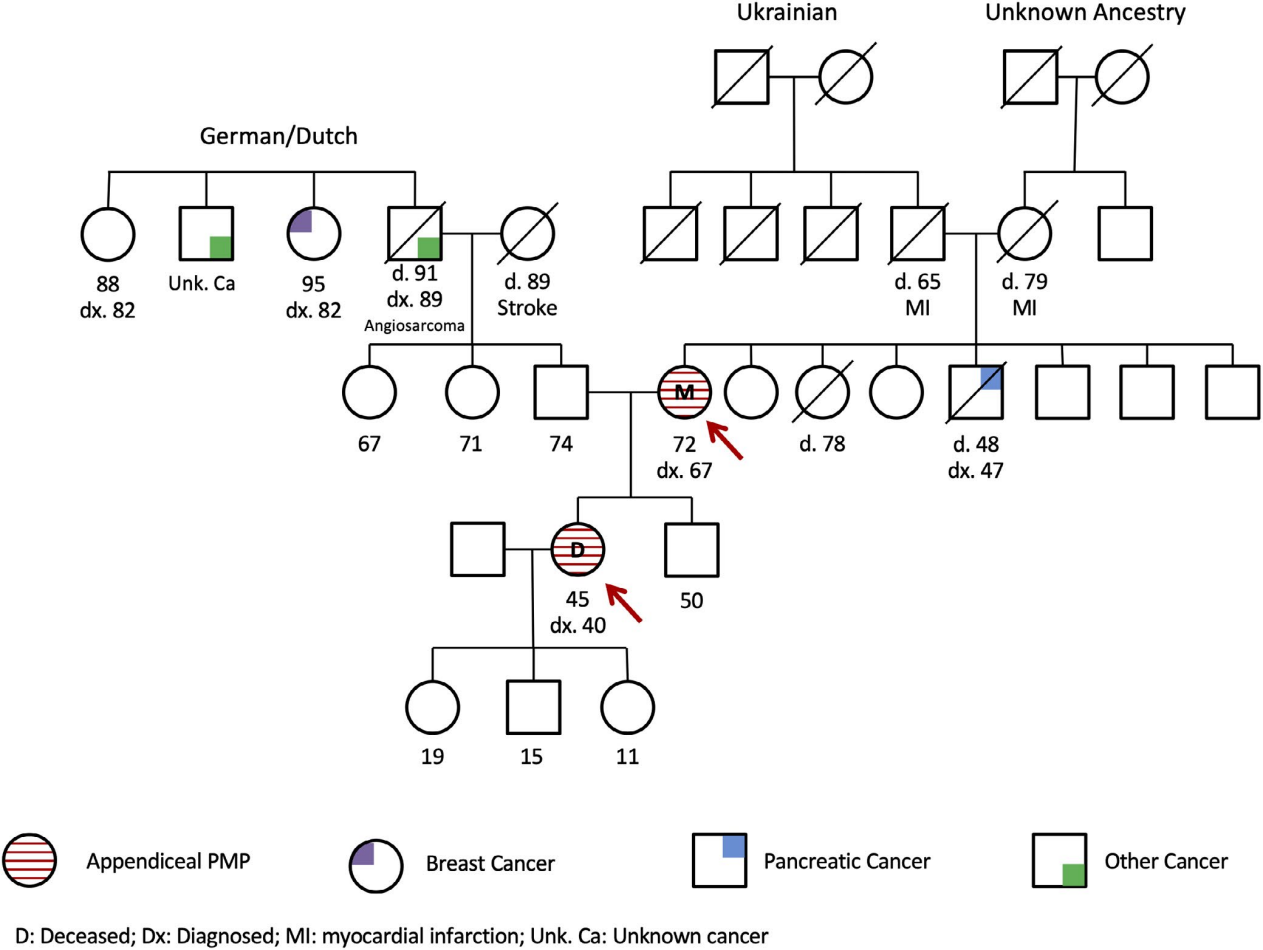
Mother/daughter family pedigree. D: Deceased, Dx: Diagnosed, MI: myocardial infarction, Unk. Ca: Unknown cancer

The mother's family cancer history is significant for a brother who was diagnosed with pancreatic cancer at age 47 (deceased age 48). The daughter's paternal family cancer history is significant for her paternal grandfather diagnosed with angiosarcoma at age 89 (deceased age 91), two paternal great aunts diagnosed with breast cancer at ages 82 and 88, and a paternal great uncle who had an unknown cancer (Figure 3).

### 4.2 Germline genetic testing

Both patients had a heterozygous pathogenic mutation in the RAD51C gene (c.955C>T) and a heterozygous likely pathogenic variant in the FH gene (c.1431_1433dupAAA).

### 4.3 NGS tumor somatic genetic testing

#### 4.3.1 Mother & daughter

Both patients had known pathogenic RAD51C (R319*) and KRAS (G12D) mutations in the appendiceal primary tumors. Both had GNAS mutations at the same position, but with different base substitutions (mother: R201H; daughter: R201C). They also shared two variants of uncertain significance, including FH (K477_N478insK) and TSC1 (H732Y) (Table 2).

**Table 2:** Genetic testing results for mother and daughter

Gene	Mother mutation	Daughter mutation	Pathway
Germline genetic testing (blood sample)			
RAD51C^a^	c.955C>T (R319*)		DNA repair pathway, homologous recombination
FH^b^	c.1431_1433dupAAA (K477_N478insK)		Fumarase formation for citric acid cycle and ATP formation
Somatic genetic testing (appendix tumor)			
RAD51C^a^	R319* (c.955C>T)		DNA repair pathway, homologous recombination
FH^c^	K477_N478insK (c.1431_1433dupAAA)		Fumarase formation for citric acid cycle and ATP formation
TSC1^c^	H732Y		Tumor suppressor function, controls cell growth and size
KRAS^a^	G12D		Cell proliferation & death, decreased sensitivity to EGFR
GNAS^a^	R201H	R201C	Activation of adenylyl cyclase & increase in cAMP for intracellular transduction
DIS3^a^	D458N	‐	RNA processing and decay
AXIN1^c^	R484C	‐	WNT pathway, formation of β‐catenin destruction complex
PRDM1^c^	A711T	‐	Repressor protein for β‐IFN gene, drive maturation of B‐lymphocytes
RB1^a^	‐	R251*	Retinoblastoma protein, known tumor suppressor & negative regulator of cell cycle
SMAD4^a^, ^d^	‐	R361H	Transcriptional regulator of TGF‐β pathway, tumor suppressor
CREBBP^c^	‐	P1010L	Transcriptional coactivator, cell cycle regulation
FANCA^c^	‐	L379V	Attracts DNA repair proteins, interacts with BRCA1
MED12^c^	‐	Q2119_Q2120insHQQQ	Mediator complex that regulates gene activity, early development (cell growth, migration, differentiation)
PIK3C2B^c^	‐	R1118C	PI3‐kinase for signaling pathways in cell proliferation, oncogenic transformation, cell survival, and migration
SMARCA4^c^	‐	A1536S	Transcription regulation, DNA repair and replication

^a^ Pathogenic.

^b^ Likely pathogenic.

^c^ Variant of Uncertain Significance.

^d^ Subclonal (MAF < 10%).

#### 4.3.2 Mother only

The mother had three additional somatic mutations, including a known pathogenic mutation in DIS3 (D458N) and two variants of uncertain significance in AXIN1 (R484C) and PRDM1 (A711T).

#### 4.3.3 Daughter only

The daughter had seven additional somatic mutations, including two known pathogenic mutations in RB1 (R251*) and SMAD4 (R361H) and five variants of uncertain significance in CREBBP (P1010L), FANCA (L379V), MED12 (Q2119_Q2120insHQQQ), PIK3C2B (R1118C), and SMARCA4 (A1536S).

## 5 DISCUSSION

We present the first description of familial appendiceal LGMCP with comprehensive genetic analysis. Appendiceal cancers are rare with an annual age‐adjusted incidence of 0.4 cases per 100 000.^24^ PMP mainly occurs in the presence of an AMN with an incidence of approximately 0.2 per 100 000 per year.^25^ Thus, the occurrence of appendiceal PMP in two first‐degree relatives raises the possibility that certain genetic factors are responsible for tumorigenesis and outcomes.

Appendiceal malignancies include a spectrum of histopathologies. In our cases, both had LGMCP originating from LAMN. Although staged like cancer with high propensity for abdominal metastases, LGMCP does not have traditional carcinoma features.^26^ Unlike higher grade appendiceal malignancies, there is little atypia, no invasive adenocarcinoma components, and lymph node and extra‐abdominal metastases are very rare.^5^ Rather, LGMCP is characterized by excessive mucin production that causes mechanical compression of abdominal organs. Contrary to its benign appearance, we found multiple oncogenic mutations in both tumors. While LGMCP has a protracted course compared to other subtypes, with 5‐year overall survival reported as high as 85%,^27^, ^28^ some patients do not respond well to standard treatment.^29^ This unique case of familial LGMCP provides the best setting to compare outcomes.

Despite similar disease presentation, both patients had factors in their treatment that could impact outcomes. First, the mother's gynecologist performed noninvasive paracentesis which provided the initial tissue diagnosis of appendiceal cancer. She was then referred immediately to a CRS/HIPEC center. The daughter, however, was taken to surgery before biopsy results were final. This resulted in an incomplete first surgery, which negatively impacts survival, increasing the time to CRS/HIPEC and difficulty of the procedure due to inflammation and adhesions.^30^, ^31^ Subsequently, the daughter's initial CRS/HIPEC attempt was aborted. When extensive peritoneal disease is present or suspected, biopsy results should be reviewed prior to extensive procedures so optimal treatment is offered upfront. Second, the mother had an incomplete cytoreduction with residual small bowel disease. Although an incomplete cytoreduction is one of the strongest predictors for poor survival, the mother still had a durable response and is clinically and radiographically disease free. This underscores the importance of HIPEC in addition to CRS in appendiceal LGMCP for ascites and symptom control. Thus, molecular factors may explain differences in their response.

Genetically, both mother and daughter shared germline mutations in RAD51C (c.955C>T), known to be pathogenic, and FH (c.1431_1433dupAAA), considered likely pathogenic. The appendiceal tumors also had matching mutations. RAD51C is involved in homologous recombination and DNA repair. This specific variant is reported in individuals with ovarian cancer and family history of breast and ovarian cancer,^32^ as well as triple negative breast cancer.^33^ FH is involved in ATP generation. This mutation is associated with autosomal recessive fumarate hydratase deficiency (FHD), which causes autosomal dominant hereditary leiomyomatosis and renal cell cancer (HLRCC).^34^ To our knowledge, this is the first case of either of these mutations reported in appendiceal cancer. Their presence in this unique case warrants further investigation in a larger population, as they could influence disease development and it raises the possibility of a genetic susceptibility to appendiceal LGMCP.

There are few reports of germline genetic testing in appendiceal tumors. Recently, Lung et al performed germline whole exome sequencing on a father/daughter pair with LGMCP and HGMCP, respectively, and identified 15 matching mutations.^22^ Although none matched our cases, this could be due to histopathologic differences. However, these sporadic cases can provide important information about disease mechanisms and need further investigation. The only other reported germline testing in familial appendiceal cancer was performed in the brother of a brother/sister pair with appendiceal mucinous adenocarcinomas, who had a variant of undetermined significance in PKD1, associated with polycystic kidney disease, and normal expression of DNA repair genes associated with Lynch syndrome (MLH1, MSH2, and MSH6).^20^ It has been hypothesized that familial Lynch syndrome and corresponding microsatellite instability (MSI) present in many right‐sided colon cancers may contribute to appendiceal cancer. However, there are no reported cases of Lynch syndrome with appendiceal cancer and low prevalence of MSI in the appendix suggests a different mechanism in this location.^35^ Consistently, both of our patients were also MSI stable.

In addition to the germline mutations, both of their appendiceal tumors harbored KRAS and GNAS mutations. KRAS mutations, known to be oncogenic and correlated with decreased sensitivity to EGFR therapies, are reported in approximately 36%‐40% of colorectal cancers and are associated with a worse prognosis in pancreatic cancers.^36^, ^37^, ^38^ KRAS mutations are also common in LGMCP (50%‐94%) and HGMCP (55%‐100%), but there is no correlation with prognosis.^17^, ^19^, ^39^ The high incidence of KRAS mutations in appendiceal cancers distinguishes them from colorectal cancers and may partially explain their limited response to systemic chemotherapies.^40^ Likewise, GNAS mutations are reported in 50%‐72% of AMN, but without correlation to clinical outcomes.^41^, ^42^, ^43^ Both patients had GNAS mutations at the same loci, but different base substitutions (mother: R201H; daughter: R201C). Borazanci et al profiled 588 appendiceal tumors and found that AMN have approximately the same incidence of GNAS mutations as intraductal papillary mucinous neoplasms, suggesting that it may play a role in disease development and mucin production, and have similar molecular profiles to pancreatic cancers.^17^ Although we are unable to determine the direct link, it is interesting that the mother's brother died of pancreatic cancer. The interaction of these mutations may account for disease presentation.

Both appendiceal tumors also harbored TSC1 missense mutations, which disrupt its tumor suppressor function. This mutation was also tested in the germline setting, but was negative. TSC1 mutations have not previously been reported in appendiceal malignancies; although, there is evidence they are likely underrepresented in the literature. Iyer et al found TSC1 mutations in the tumor genome of patients with metastatic bladder cancer who had a durable and significant response to everolimus, an mTOR inhibitor.^44^ An everolimus basket trial also showed TSC1 mutations conferred clinical benefit.^45^ The daughter also had a somatic mutation in PIK3C2B, a member of the mTOR pathway, further suggesting the importance of this pathway in LGMCP and warranting further investigation. NGS of appendiceal tumors can identify actionable targets and provide other treatment options, such as everolimus, which may be more effective than traditional colon cancer regimens.

Despite matching disease presentation and five mutations, the patients had different treatment response and clinical outcomes. Whereas the mother has no clinical evidence of disease and has been asymptomatic for 4.8 years despite an incomplete cytoreduction, the daughter failed two attempts at complete CRS/HIPEC and multiple lines of chemotherapy. The unique somatic mutations harbored in their appendiceal tumors could provide valuable insight for treatment response. The mother had three (one pathogenic and two variants of uncertain significance) and the daughter had seven (two pathogenic and five variants of uncertain significance) additional somatic mutations. In some cancers, such as lung and colorectal adenocarcinomas, high mutational burden is associated with decreased survival, which could explain the differences in outcomes.^46^, ^47^ However, each mutation impacts outcomes differently, with decreased survival associated with more designated driver mutations than passenger mutations.^47^ Identifying driver vs passenger mutations is essential to understand LGMCP pathogenesis.

Interestingly, both patients harbored somatic mutations associated with the transforming growth factor‐beta (TGF‐β) and wingless‐related integration site (WNT) canonical pathways, which play important roles in regulating cell growth, differentiation, and homeostasis. The daughter harbored a pathogenic mutation in SMAD4, associated with high cytologic grade, high cellularity, destructive invasion, and decreased overall survival in pancreatic and colorectal cancers.^48^ Other studies also reported dysregulation of the TGF‐β pathway in PMP.^42^, ^49^, ^50^, ^51^, ^52^ The mother had a mutation in AXIN1, a component of the β‐catenin destruction complex of the WNT canonical pathway that prevents β‐catenin from accumulating in the nucleus to regulate proliferation. Previously, we showed that treatment with antibiotics altered the expression and localization of β‐catenin in HGMCP.^14^ Other studies reported WNT pathway mutations in mucinous appendiceal neoplasms at a similar incidence to colorectal cancer.^50^ The TGF‐β SMADs and WNT mediator β‐catenin work together to control gene expression in the nucleus^53^, ^54^, ^55^ and a dysfunctional interaction of these pathways may play a role in PMP pathogenesis.

Overall, familial appendiceal tumors with PMP are extremely rare. We present the first instance of LGMCP originating from LAMN in two first‐degree relatives. To our knowledge, this is the fifth report of familial appendiceal neoplasms, and the third report of first‐degree relatives with appendiceal cancer: a father/daughter with appendiceal carcinoid neoplasms, identical twin brothers with mucinous adenomas, a brother and sister with mucinous adenocarcinoma, and a father/daughter with LGMCP and HGMCP, respectively.^16^, ^20^, ^21^, ^22^ By evaluating both germline and somatic genetic factors, these cases may elucidate the molecular factors that contribute to and guide further investigation of appendiceal cancer and treatment response in larger cohorts. Identifying differences in molecular alterations between exceptional and poor responders in a larger cohort may elucidate targeted therapies and additional treatment options.

There are several lessons that can be learned from this case. First, it highlights the importance of early referrals to a HIPEC center. The mother was effectively treated with upfront CRS/HIPEC because of early tissue diagnosis, while the daughter first underwent an incomplete debulking by a different specialty, leading to a more difficult CRS/HIPEC that was aborted. Second, hereditary appendiceal cancer is possible, especially with the discovery of these novel pathogenic mutations in RAD51C and FH. Defining this link could have resulted in earlier diagnosis for the daughter and in the future could be prevented for others. Third, differences in somatic mutations may account for the exceptional and poor response seen in these cases. Despite shared family history, clinical presentation and diagnosis, two germline mutations, and three somatic mutations, the mother responded well to CRS/HIPEC, even with an incomplete cytoreduction, while the daughter has been unable to achieve a durable response. Moreover, appendiceal malignancies differ from colorectal cancers and may not respond to the same agents, as seen with the daughters many failed lines of 5FU‐based chemotherapy. Somatic tumor testing may provide additional treatment options for appendiceal LGMCP.

## CONFLICT OF INTEREST

The authors have declared no conflict of interest.

## AUTHOR CONTRIBUTIONS

Mary Caitlin King, BS: involved in conceptualization, data curation, formal analysis, investigation, writing‐original draft, and writing‐review and editing. Carlos Munoz‐Zuluaga, MD: involved in conceptualization, formal analysis, investigation, and writing‐review and editing. Panayotis Ledakis, MD and Armando Sardi, MD: involved in conceptualization, investigation, supervision, and writing‐review and editing. Kimberley Studeman, MD and Vadim Gushchin, MD: involved in investigation, supervision and, writing‐review and editing. Michelle Sittig, RN: involved in writing‐review and editing].

## ETHICAL APPROVAL

This study was approved by the IRB, and all patients gave preoperative consent.

## CONSENT STATEMENT

Published with written consent of the patient.
